# Characterization of placental and decidual cell development in early pregnancy loss by single-cell RNA sequencing

**DOI:** 10.1186/s13578-022-00904-5

**Published:** 2022-10-08

**Authors:** Yuhua Zheng, Jing Pan, Chenglai Xia, Haiying Chen, Huadong Zhou, Weina Ju, Jerzy Wegiel, Leslie Myatt, James M. Roberts, Xiaoling Guo, Nanbert Zhong

**Affiliations:** 1 Maternity and Child Healthcare Hospital, Foshan Women and Children, 11 W. Renmin Lu, Foshan, 528000 China; 2 New York State Institute for Basic Research in Developmental Disabilities, 1050 Forest Hill Road, Staten Island, NY 10314 USA; 3 Oregon Health and Science University, 3181 SW Sam Jackson Park Rd, Portland, OR 97239 USA; 4 Department of Obstetrics, Gynecology and Reproductive Sciences, Epidemiology and Clinical and Translational Research University of Pittsburgh, Magee-Womens Research Institute, 204 Craft Avenue, Pittsburgh, PA 15213 USA

**Keywords:** Single-cell RNA sequencing, Miscarriage, First trimester, Pregnancy, Transcriptomics

## Abstract

**Background:**

Early pregnancy loss (EPL) presents as sporadic or recurrent miscarriage during the first trimester. In addition to chromosomal defects, EPL may result from impairment of the placental-decidual interface at early gestational age due to gene-environmental interactions.

**Methods:**

To better understand the pathogenesis associated with this impairment, cell development in chorionic villi and decidua of different forms of EPL (sporadic or recurrent) was investigated with single-cell RNA sequencing and compared to that of normal first-trimester tissue.

**Results:**

Unique gene expression signatures were obtained for the different forms of EPL and for normal tissue and the composition of placental and decidual cell clusters in each form was established. In particular, the involvement of macrophages in the EPL phenotypes was identified revealing an immunoactive state.

**Conclusion:**

Differential gene expression and unique marker genes among cell clusters from chorionic villi and decidua of miscarried and normal pregnancies, may lead to identification of biomarker for EPL.

**Supplementary Information:**

The online version contains supplementary material available at 10.1186/s13578-022-00904-5.

## Background

Early pregnancy loss (EPL), which includes sporadic miscarriage or recurrent miscarriage, is the loss of a pregnancy during the first 13 weeks of gestation and accounts for about 10–15% of all clinically recognized pregnancies [1, 2]. Sporadic miscarriage primarily represents failure of abnormal embryo/fetus to progress to viability, with about one-half of cases resulting from chromosomal abnormalities, mainly aneuploidies, of the embryo [3–5]. Recurrent miscarriage, the loss of a minimum of two consecutive pregnancies, may be the result of abnormal endometrial and/or placental development that occurs in about 1–3% of EPLs and may involve maternally driven causes [6, 7]. Genetic-environmental interactions, including genetic predisposition [8], have been determined in more than 100 gene loci in addition to fetal chromosomal aberrations that may identify 29–60% of cases of recurrent miscarriage [6, 8]. Maternal hormones or metabolism, uterine anatomy, infection, occupational and personal habits, thrombophilia, immune disorders, or environmental exposure have also been reported as possible multifactorial backgrounds for EPL. Epigenetic dysregulation in pregnancy may play a role in the decidualized endometrium, acting as a biosensor of embryo quality, which if disrupted, may lead to implantation of embryos destined to miscarry [6, 9].

Many studies have focused on early development and function of the placenta, the maternal fetal interface [10, 11]. Various subtypes of placental cells have been identified [12–14] that are derived from the embryonic trophectoderm and extraembryonic mesoderm to compose the placental structure [15, 16]. Three main epithelial trophoblast types, the cytotrophoblast (CTB), syncytiotrophoblast (STB), and extravillous trophoblast (EVT), are characterized in placental tissues delivered at term [13, 17, 18]. The CTBs, also termed villous cytotrophoblasts (VCTs), form a single layer surrounding the villous stromal cell core and serve as the source of the epithelial “stem cells” of the placenta, which differentiate into either STB or EVT. The STB is a multinucleated cell layer continuously renewed by CTB fusion into it that covers the entire surface of the villous tree throughout pregnancy to secrete the hormones necessary for pregnancy maintenance and to form a barrier across which nutrient and gas exchange can take place. The villous core consists of fibroblasts (FBs); Hofbauer (HB) cells, which are placental villous macrophages of fetal origin that are present throughout pregnancy; and capillary networks. The EVTs, characterized by the expression of *HLA-G*, *DIO2*, *LAIR2*, *HTRA4*, and *MFAP5*, erupt from the tips of the placental villi, proliferate, and differentiate to form a trophoblast cell column and to invade maternal decidua. The bulk of EVT differentiation occurs during the first trimester, before the onset of maternal arterial blood flow into the intervillous space of the placenta. EVTs anchor the placenta to the uterine wall and remodel maternal spiral arterioles to provide ample blood supply to the growing fetus. A subset of EVTs may be involved in remodeling of maternal spiral arteries and uterine glands [13, 18–21]. The fetal macrophages (Hofbauer cells) can be detected in the placental villous stroma as early as 3 weeks post-conception and are present throughout pregnancy. They are likely to have a variety of functions including control of villous remodeling and differentiation, hormonal secretion, and trophoblast turnover [22, 23]. Several lines of evidence have led to the postulation that HB cells may play a role in infection during pregnancy, and they are believed to be increased in EPL as results of their divergent roles in immunity and inflammation [23, 24].

During the implantation window, the endometrium becomes poised to transition to a pregnancy state, a process driven by differentiation of stromal cells into decidual cells (DCs). Cells in the decidua include perivascular (PV) cells, stromal (dS) cells, decidual natural killer (dNK) cells, and decidual macrophages (dM) [25]. Two subtypes of PV cells, the PV1 and PV2, were identified to share smooth muscle marker *MGP*. However, PV1 and PV2 have a higher expression level of *MCAM* and *MMP11*, respectively. Three subtypes of dS cells were characterized to express *DKK1*, through which trophoblast cell differentiation was determined to be downregulated by *HoxB7* gene [26]. dS1 cells share the expression of *ACTA2* and *TAGLN* with PVs but lack expression of the classic decidual markers *PRL* and *IGFBP1*. Both dS2 and dS3 express *IGFBP1, IGFBP2*, and *IGFBP6* and share markers with decidualized stromal cells [27]. The dS3 subset expresses *PRL* and *CYP11A1* [25]. Recently, the NK cells dNKp and dNK1- dNK4 were documented to accumulate in recurrent miscarriage [28]. In this study, we applied single-cell RNA sequencing (scRNA-seq) to test the hypothesis that differential gene expression profiles may present as cell-specific in different tissues taken from patients with EPL, either sporadic or recurrent, compared to tissue from elective termination of unplanned pregnancies (ET).

## Materials and methods

### Ethics statement

All experiments involving human specimens including placentas were conducted according to the ethical policies and procedures. The research design, along with written informed consent obtained from the participating subjects, of this project was reviewed and approved by the Ethics Committee of Foshan Maternity and Child Healthcare Hospital (FSFY-MEC-2020-030).

### EPL and ET

Five individual patients were recruited for each group of A, B, C, and D, for whom demographic information of EPL and ET is summarized in Table 1. Group A comprised early missed sporadic miscarriage without a fetal heartbeat detectable by ultrasound when the patient presented at the prenatal clinic. Group B were missed sporadic miscarriage with a detectable fetal heartbeat when the patient sought to see a doctor in the clinic, but with subsequent loss of the fetal heartbeat. Group C referred to recurrent miscarriage for two or more pregnancies. Group D was the control group of normal pregnancies with elective termination of pregnancy. The entire chorionic villi and gestational sac containing decidua were collected via vacuum aspiration. The tissues were immediately washed and dissected in cold phosphate-buffered saline (PBS) until no blood clots were visible, followed by soaking and maintenance in cold MACS Tissue Storage Solution (Miltenyi Biotec, Waltham, MA) for tissue transfer to the research laboratory (within two hours). The tissues were digested with a mixture of proteases containing 0.125% trypsin, 0.05% type IV collagenase, and 0.04% DNase (Sigma-Aldrich, St. Louis, MO) that were diluted in Dulbecco’s modified eagle medium (DMEM, HyClone, Logan, UT) at 37 °C for an initial 7 min, followed by washing with DMEM three times, and then a second period of digestion of 20 min. Cells were passed through a Falcon 40-μm cell strainer (#352,340, Corning, Oneonta, NY), precipitated by centrifugation at 300*g* for 5 min after the red blood cells were lysed, resuspended in PBS, and counted with trypan blue [17]. The average cell counts and cell viability (counts × 10,000)/viability %) of the four groups were 33.9/89.8, 52.4/86.6, 73.6/88, and 38.1/83, respectively (Table 1), which met the requirement of scRNA-seq platform with 10 × Genomics technology (www.10xgenomics.com).

**Table 1:** Demography of patient groups

Sample label	Age (yrs.)	Days of gestation by LMP	Gestational age (weeks)	Length of embryo (mm)	BMI	Education	Cell recovered (× 10,000)	Cell viability (%)
A1	30	41	6 +	4	29.7	College	7.5	84
A2	26	72	7	5	18.2	High school	17	85
A3	32	68	6 +	3	24.0	College	80	94
A7	35	81	6	< 1*	23.6	College	35	93
A8	33	88	6 +	3	24.2	UN	30	93
Average	31.2	70	6.5	3.75	23.9		33.9	89.8
B1	29	75	6	2	19.5	College	48	91
B2	29	68	7	5	19.1	College	23	85
B4	25	74	6 +	5	18.0	UN	90	92
B5	28	68	7	5	20.5	Junior high	23	82
B8	22	69	6 +	2	17.0	UN	128	83
Average	26.6	70.8	6.7	3.8	18.8		62.4	86.6
C1	33	71	6 +	2	19.1	UN	120	92
C2	30	77	6 +	4	20.1	UN	13	87
C3	31	71	6 +	< 1*	22.6	College	66	83
C4	30	72	6 +	4	21.6	College	40	88
C7	26	76	6 +	4	18.7	College	129	90
Average	30	73.4	6 +	3.5	20.4		73.6	88
D1	30	48	6	< 1*	24.8	College	4.5	84
D8	23	51	6	< 1*	28.2	UN	12	85
D9	26	47	6	3	18.1	College	11	85
D10	31	49	6 +	< 1*	18.4	UN	64	97
D13	29	43	6	4	20.0	College	99	64
Average	27.8	47.6	6	3.5	21.9		38.1	83

*EPL* early pregnancy loss, *ET* elective termination of unplanned pregnancy, *LMP* last menstrual period, *UN* unknown, *BMI* body mass index

Patient groups: A: EPL of sporadic miscarriage—no fetal heartbeat in prenatal clinic; B: EPL of sporadic miscarriage—fetal heartbeat initially detected; C: EPL of recurrent miscarriage; D: ET

^*^ Length of embryo (mm) < 1 indicates that the embryo signal detected by ultrasound examination was not quite clear

### scRNA-seq and data process

The Chromium Single Cell 3’ Reagent Kit (10 × Genomics) was applied to scRNA-seq. The procedure was performed according to the manufacturer’s instructions. The library was constructed and sequenced on Illumina PE150 platform with 26 bp (Read1) and 98 bp (Read2) [13, 29]. The raw data were processed by using CellRanger V3.0.2 (https://support.10xGenomics.com/single-cell-expression/software) [30], followed by using Seurat V3.0 (https://satijalab.org/seurat/) for cell clustering, gene expression, and marker genes, trajectory pseudotime with Monocle (a tool for quantification of expressed mRNA in scRNA-seq data) [31], gene module with WGCNA (Weighted Gene Co-Expression Network Analysis) [32], cell cycle with Cyclone, cell–cell interactions with Scanpy V1.4.6, gene interaction with Cytoscape V3.1.0 (http://cytoscape.org), and cell network with String (https://string-db.org/) [33–35]. PCA and UMAP (uniform manifold approximation and projection) were applied for data visualization [36]. Gene differential expression, gene ontology functional annotation, and pathways that associated with specific cell cluster were analyzed with DAVID (https://david.ncifcrf.gov/) [37] and compared to the online database of KEGG (http://www.kegg.jp/kegg) and Gene Orthology (http://geneontology.org), as we described previously [38].

### Statistical analysis

The ImageJ software (https://imagej.en.softonic.com) was used to calculate relative expressions. Comparison of cell clusters among four groups was analyzed with SPSS 17.0 (http://software.broadinstitute.org/gsea/index.jsp). GraphPad Prism Version 7.0 (GraphPad Software, San Diego, CA) was used for statistical analyses. Data were expressed as mean ± SEM values. Student t test was used for comparison between two groups, and one-way ANOVA was used for comparison between multiple groups. The difference is considered statistically significant when P < 0.05 unless otherwise stated.

### Data availability

Raw and processed data from this study have been submitted to the NCBI Gene Expression Omnibus (GEO; https://www.ncbi. nlm.nih.gov/geo) under accession numbers GSE174399.

## Results

### Differentially expressed cell clusters in the placental tissues of EPL and ET

**Fig. 1:**
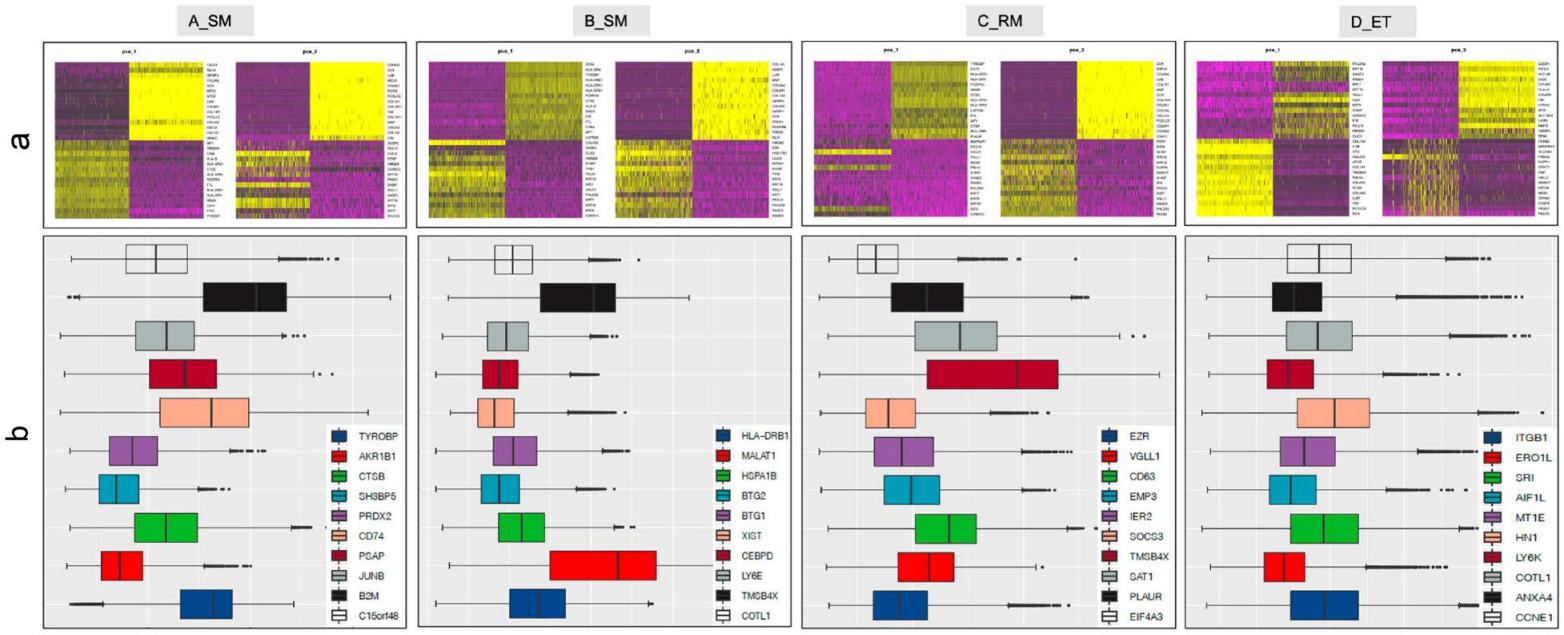
Grouping of clinically classified early pregnancy loss (EPL).** a** PCA heatmap of marker genes in cell clusters of different groups of EPL (up panel). A_SM: early missed sporadic miscarriage without intrauterine fetal heartbeat, B_SM: early missed sporadic miscarriage with intrauterine fetal heartbeat, C_RM: recurrent miscarriage, and D_ET: elective termination of unplanned pregnancy. ** b** Boxplot of highly expressed top-10 gene markers used for distinguishing groups **A**, **B**, **C**, **D** (lower panel)

EPL is a complex condition that results from genetic predisposition, epigenetic mis-regulation, immune reaction to infection and inflammation, endocrine dysfunction and imbalance of hormone, and uterine structural and endometrial abnormalities [39–41]. These etiological factors would influence placentation or decidualization at the early stage of pregnancy [42–44]. Previously, we performed a transcriptomic study on embryonic placental tissues obtained at termination of pregnancy and documented that long non-coding RNA (lncRNA) played an epigenetic regulated role on infection and inflammation pathways that are associated with pregnancy loss [45]. In the current study, cell clusters were classified into villi-, decidua-, and immune- categories from placental tissues of EPL and ET, based on previously described placental cell clusters [13, 17, 25]. Proportions and subtypes of these cell clusters were determined from embryonic placental tissues that were derived from 20 pregnancies at 6–7 gestational weeks, with five pregnancies in each of four groups A, B, C, or D, respectively (Table 1). These four groups could be distinguished at the molecular level with highly expressed genes, as presented in Fig. 1. A total of 217,649 genes, including 40,705 from group A, 51,506 from B, 43,755 from C, and 75,683 from D, were sequenced.

**Fig. 2:**
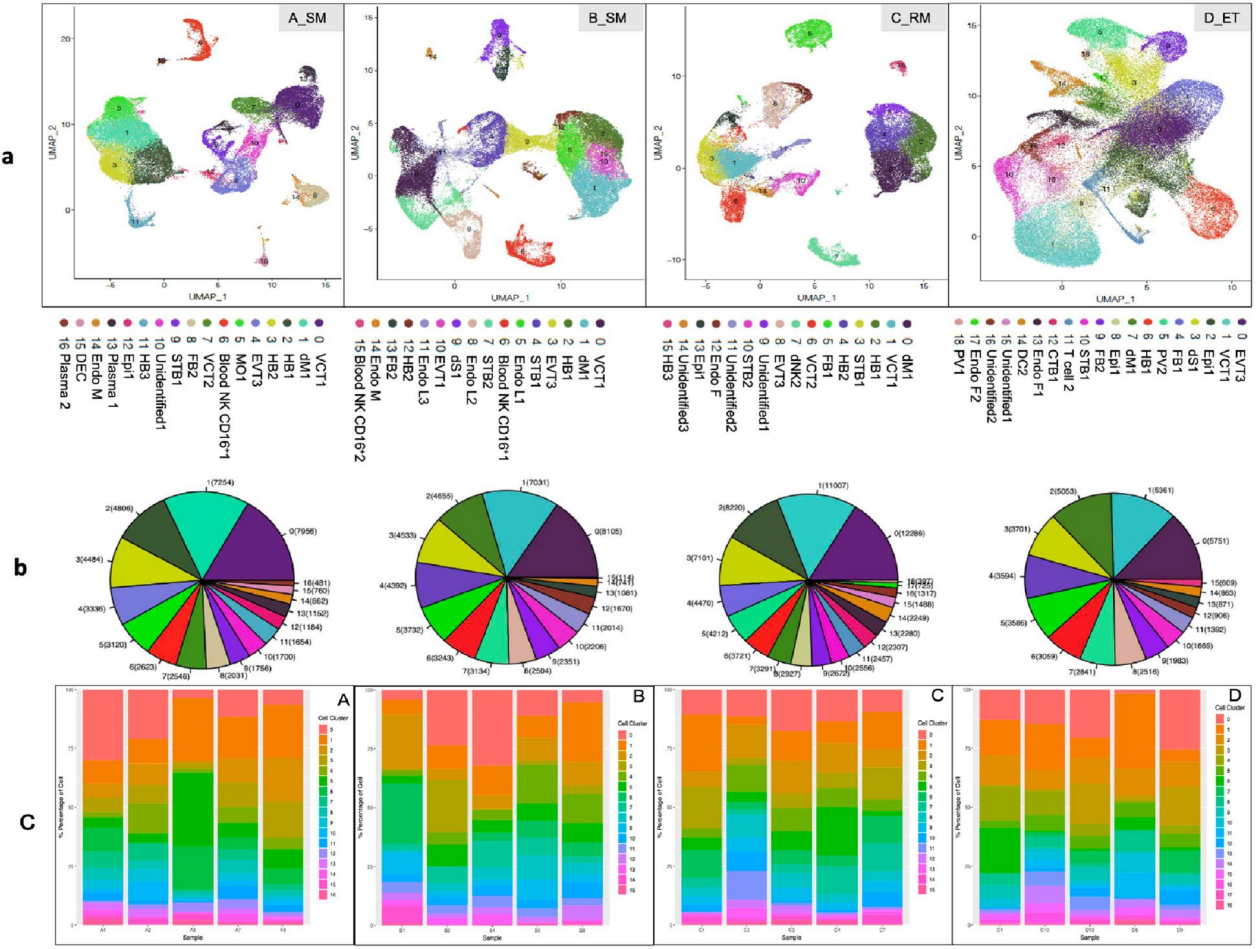
Annotation of scRNA-seq in early pregnancy loss. **a** UMAP of scRNA-seq data from the four early pregnancy loss groups A_SM, B_SM, C_RM, and D_ET. Each color presents a cell cluster of a different cell type. **b** Number of cells (shown in brackets) that have been sequenced for each cluster (color and number corresponding to the UMAP). **c** Percentage (%) of cells (Y axis) in each cluster for individual placental samples in groups A, B, C, and D (X axis). *CTB* cytotrophoblasts, *VCT* villous cytotrophoblasts, *STB* syncytiotrophoblasts, *EVT* extravillous trophoblasts, *HB* Hofbauer (fetal macrophage) cells, *FB* fibroblasts, *MO* monocytes, *Plasma* plasma cells, *Blood NK CD16*^*+*^ blood natural killer CD16^+^ cells, *DEC* decidual cells, *dM* decidual macrophages, *dNK* decidual natural killer cells, *dS* decidual stromal cells, *DC* dendritic cells, *PV* perivascular cells, *Epi* epithelial glandular cells, *Endo M* endothelial maternal cells, *Endo F* endothelial fetal cells, *Endo L* endothelial lymphatic cells.

After groups A, B, C, and D were differentiated with highly expressed markers, which were characterized by principal component analysis (PCA), that specifically associated with the clinically identified phenotypes, placental and decidual cell clusters were identified with previously reported gene markers for each cell cluster [13, 23], followed by characterization of single-cell gene expression profiles. Group D with normal placental tissues that were obtained by elective termination was used as a control, to which tissues of groups A, B, and C were compared. Both groups A and B comprised sporadic miscarriages, yet the period of intrauterine development of placental cells in group B could be longer than in group A, indicating that the sign of intrauterine life lasted longer before pregnancy was terminated, although both groups had a similar gestational age. Indeed, the detailed cell clusters differed from each other among these four groups of pathologic pregnancies (Fig. 2). For example, CTB1, a subtype of very early VCT1, was identified from group D but not in EPL, and VCT2 was seen in group A (as well as group C), but not in group B (nor in group D). These findings suggested the difference in trophoblast differentiation and development. The developmental difference of placenta occurred not only in trophoblasts, but also in DC clusters. PV1, PV2, dS1, and DC2 have been identified in group D only, and plasma cell cluster was not present in either EPL or ET, except in group A (cluster 13 and 16).

### Three most abundant cell clusters in EPL

**Fig. 3:**
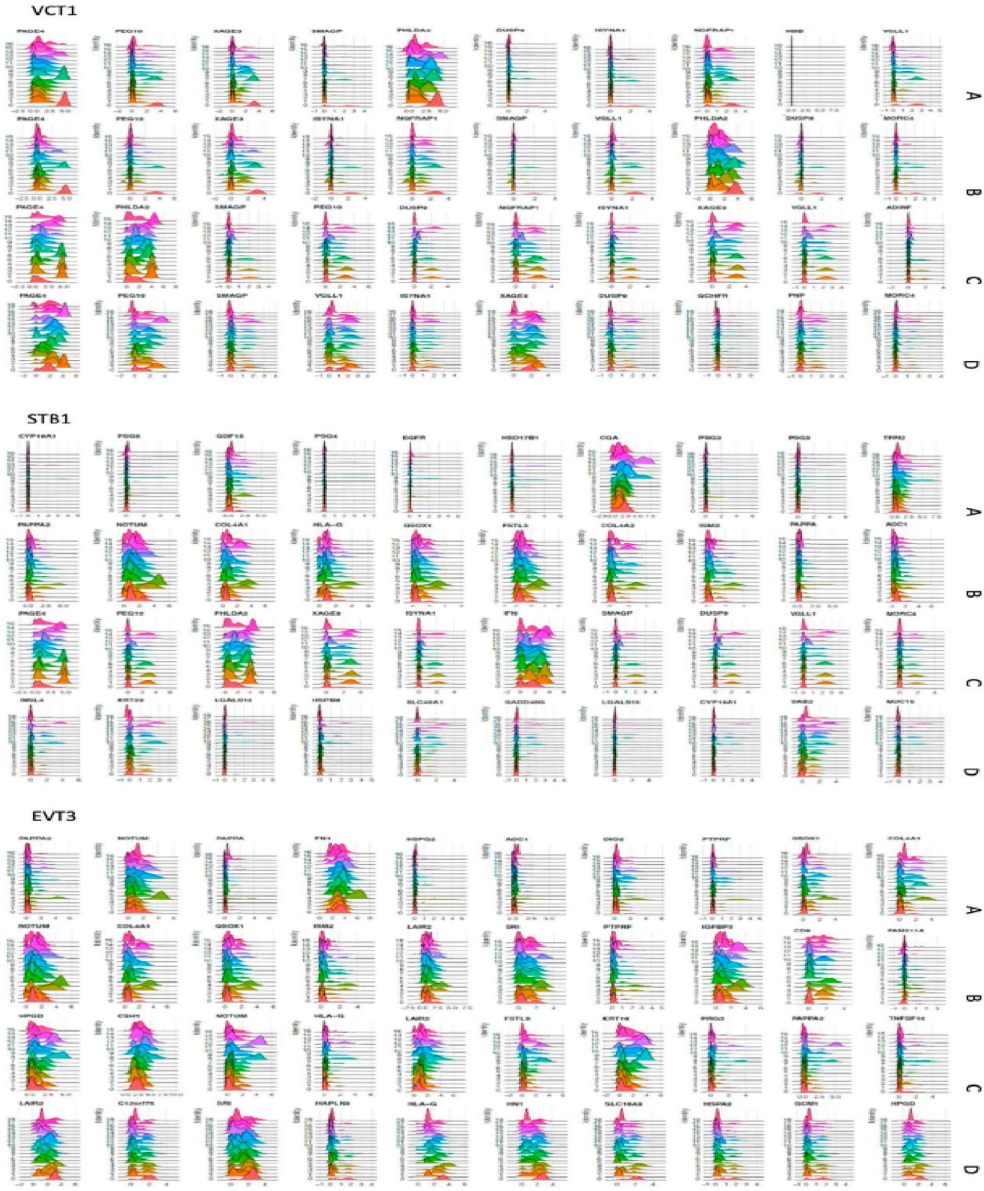
RidgePlot of top-10 marker genes for trophoblast clusters VCT1, STB1, and EVT3. Top-10 marker genes, which were expressed in EPL groups A_SM, B_SM, C_RM, and in ET group D_ET, are presented for trophoblast cell cluster VCT1 (up), STB1 (middle), and EVT3 (lower). The marker genes are shown at the top of RidgePlot

The three most abundant cell clusters in EPL were VCT1, dM1, and HB cell (HB1), which were different from EVT3, VCT1, and epithelial glandular 1 cell (Epi1) in ET (Fig. 3). VCT1 accounted for 16.68%, 15.74%, 12.25%, and 14.54% in groups A, B, C, and D, respectively; dM1 accounted for 15.21%, 13.65%, 13.14%, and 4.35% in groups A, B, C, and D, respectively; and HB1, for 10.07%, 9.04%, 11.55%, and 4.92% in groups A, B, C, and D, respectively, in EPL. However, EVT3 accounted for 6.99%, 8.80%, 5.75%, and 16.23% in groups A, B, C, and D, respectively, in ET (Fig. 2b). These data clearly demonstrate that the presence of macrophages, either dM1 or villous HB1, as the major cell cluster in EPL, strongly indicate that immunoreaction has been an etiological factor underlying the pathogenic process of EPL, in either sporadic or recurrent miscarriage (p < 0.001). The finding of VCT1 as the leading cell cluster in both groups A and B, rather than EVT as seen in group D, suggested that the transformation of VCT to EVT in SMs during trophoblast development was delayed (p < 0.005). This hypothesis was also supported by the differences of either the marker genes among groups of EPL and ET or the gene expression profiles in each cell cluster (Additional file 1: Fig. S1).

### Unique marker genes in trophoblasts may lead to identification of a biomarker for EPL

Single-cell transcriptomics has been used to describe the diverse trophoblast subtypes from peri-implantation conceptuses through placentas delivered at term [12–14, 17, 46, 47]. In this study, the single-cell–based transcriptomic expression of trophoblasts was profiled in EPL and ET, which is presented with RidgePlots (Fig. 3) and DotPlots (Additional file 1: Fig. S2). Among the top-10–expressed marker genes in VCT cell clusters, seven genes (*PAGE4*, *PEG10*, *SMAGP*, *VGLL1*, *ISYNA1*, *XAGE3*, and *DUSP9*) were present in all EPL and in ET. An additional two genes, *PHLDA2* and *NGFRAP1*, were only present in EPL, not in ET. Three genes, *HBB*, *MORC4*, and *ADIRF*, were found to be specifically expressed in group A, B, and C, respectively. *MORC4* was also found in group D, along with two unique genes, *GCHFR* and *PNP* (Fig. 3). The top-10 marker genes expressed in STB cluster cells were highly unique and specific to each group. Other than *CYP19A1*, which was expressed in group A and in group D, all marker genes were unique in each specific EPL group. Documentation of HLA-G as a marker for EVT cells [48] has been replicated in groups C and D, but not in groups A and B. Several genes showed specific expression in EVT cells of variant EPL: *NUTOM* was present in EPL, but not in ET; *QSOX1* and *COL4A1* were in both groups A and B, but not in group C; *LAIR2* was present in groups B and C; and *PAPPA2* in groups A and C. These unique or specifically expressed marker genes may lead to further investigation for biomarkers and be applied in clinical prediction of EPL.

### Unique marker genes in macrophages

**Fig. 4:**
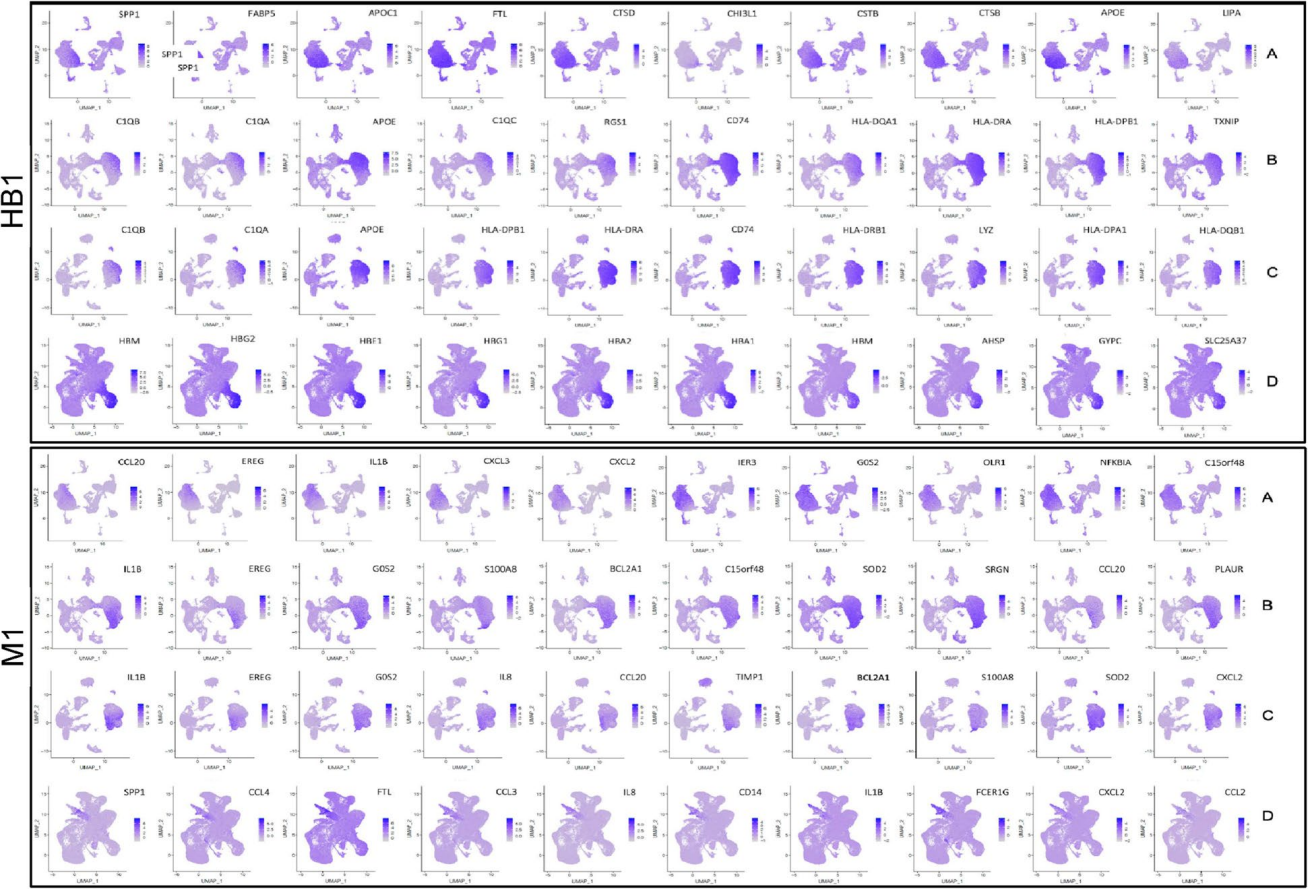
Top-10 marker genes for macrophage clusters HB1 and dM1. FeaturePlot presents top-10 marker genes that were highly expressed in cell cluster HB1 or cluster dM1 among groups A_SM, B_SM, C_RM, and D_ET

Macrophages in the early stage of placentation are composed of fetal macrophages, HBs, and dM1 (Fig. 4). Knowledge about HB cell function in EPL is limited, although HBs are important for a successful pregnancy, including placental morphogenesis, immune regulation, control of stromal water content, and the transfer of ions and serum proteins across the maternal–fetal barrier. Four types of HBs have been reported, grouped on the basis of whether or not these cells had lamellipodia, funnel-like structures, blebs, and microplicae [49], and their levels were found to be significantly higher in missed abortion cases [50]. Three subtypes of HB were identified in the current study. *ApoE* was found to be among the top-10–expressed genes in EPL but not in ET. Specifically, *ApoC1*, along with *SPP1*, *FABP5*, *FTL*, *CTSD*, *CHI3L1*, *CSTB*, *CTSB*, and LIPA, is only present in the HB1 of A_SM. HB1 is characterized by major histocompatibility complex class II (MHCII) molecules, the HLA genes; *HLA-DRA*, *HLA-DPB1*, and *HLA-DQA1* are present in group B, and *HLA-DRA*, *HLA-DPB1*, *HLA-DRB1*, *HLA-DPA1*, and *HLA-DQB1* in group C. For the HB1 cluster of the control group D, the majority of genes expressed were related to red blood cells. Seven hemoglobin subunits (*HBA1*, *HBA2*, *HBE1*, *HBG1*, *BG2*, *HBM*, and *HBZ*), one hemoglobin-stabilizing protein *AHSP*, and *SCL25A37*, which is a mitochondrial iron transporter that specifically mediates iron uptake in developing erythroid cells and plays an essential role in heme biosynthesis, were the most highly expressed genes (Fig. 4). Two newly discovered HB cell clusters, HB2 and HB3, that were not described in the earlier reports of scRNA-seq study [14, 25], were newly identified in groups A and C of the current study. HB2 was present in groups A, B, and C, and HB3 in groups A and C. Variant gene expression in the subtypes of HB cluster appear to have a pathophysiological role in the variant phenotypes of EPL. In the dM1, transcripts CCL2, CCL3, and CCL4, which are macrophage-inflammatory proteins, were present in group D only, whereas CCL20 was in the three groups of EPL but not in group D. CXCL2 was present in groups A, C, and D but not in B. These cytokine-chemokine molecules, along with superoxide dismutase 2 (SOD2), which was found in the dM1 clusters of groups B and C, supported our recently reported finding that cytokine-cytokine receptor interaction was the common pathway in spontaneous miscarriage [51].

### NK cell clusters in EPL but not in ET

**Fig. 5:**
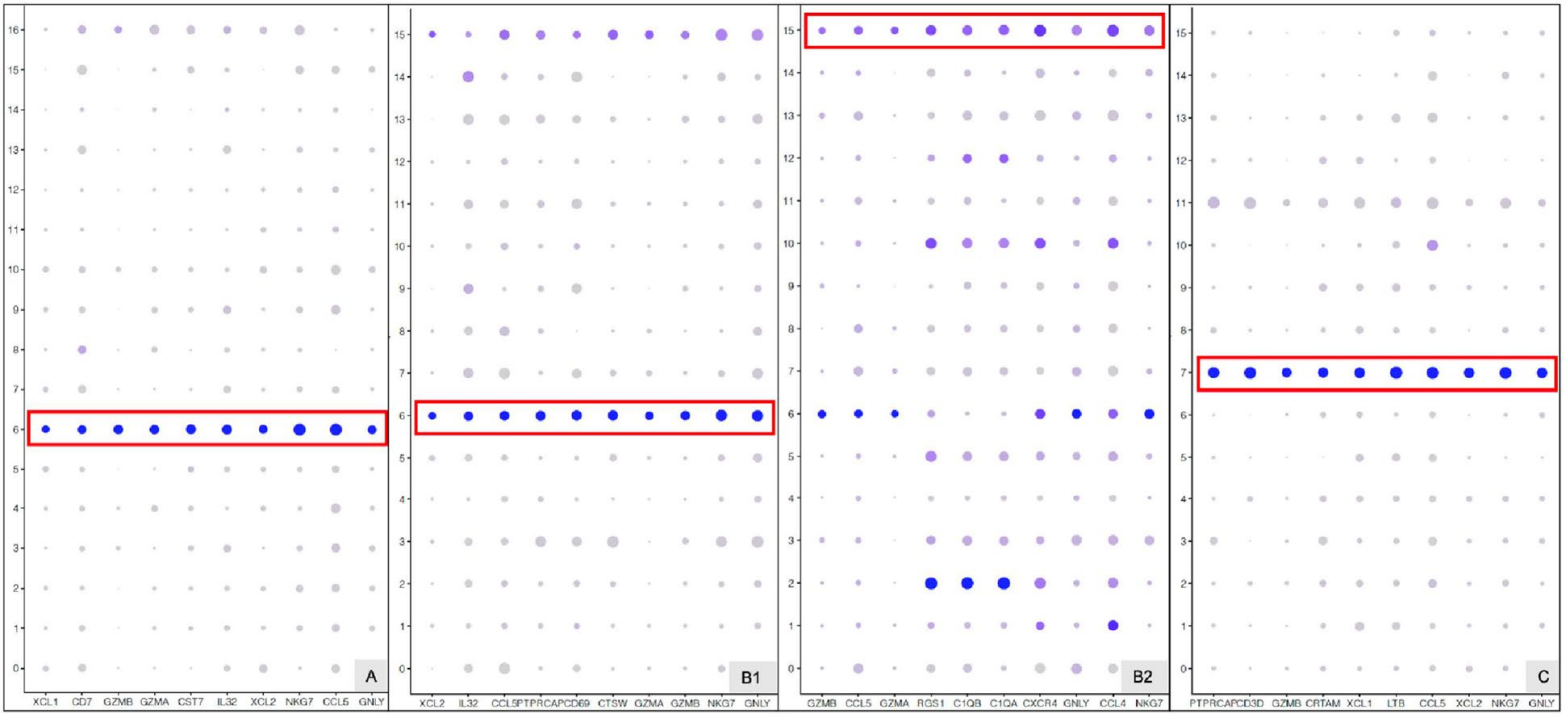
DotPlot of top-10 marker genes in NK cell clusters. Red box presents marker genes specifically expressed in NK cell clusters. Blood NK CD16^+^ cell clusters were identified in EPL A_SM or B_SM. B1 and B2 represent two subtypes of blood NK CD16^+^ cell clusters. Decidual NK cell cluster (dNK) was identified in group C_RM only

NK-CD16^+^ cell clusters were identified in groups A and B, and decidual NK2 (dNK2), in group C. No NK cell cluster was present in group D. All groups of EPL expressed genes *NKG7*, *CCL5*, *GNLY*, and *GZMB* in their NK cell clusters. The gene *GZMA*, however, was only in the clusters of groups A and B but not in C, which could be used as a good marker to distinguish SM from RM (Fig. 5). In addition, XCL2, the X-C motif chemokine ligand 2, which is a member of the C-chemokine superfamily and functions in inflammatory and immunological responses, inducing leukocyte migration and activation [52], could be another marker for EPL. Recently, three developmental branches in which dNKp differentiates into dNK1 cells (Path 1) and into two distinct branches of dNK2 and dNK3 cells (Path 2 and Path 3) have been proposed with high-resolution pseudotime prediction [53]. In addition, we applied pseudotime prediction and characterized the NK differential trajectory of these three NK clusters in groups A, B, and C (Fig. 5). The differential trajectory showed a similar pattern for NK-CD16^+^ cell clusters, although six differential states were predicted through three branching points, and eight differential states were generated from four branching points in groups A and B. Unlike groups A and B, in which the branching points 1 and 2 were relatively close, differentiation of dNK2 in group C had distant branching points between each other, which made it possible to easily distinguish the differential states.

### Other cell clusters in EPL

Cell clusters identified in EPL, other than those mentioned above, were FB1 and FB2; Epi 1 and Epi 2; endothelial cells (Endo) of maternal (Endo M), fetal (Endo F), or lymphocyte (Endo L) origin; T cells; plasma cells; monocytes (MO); dendritic cells (DCs); dS1 cells; PV1; and PV2 cells, and at least three unidentified cell clusters (Fig. 2). Among these clusters, PV1, PV2, DC, and T cells were present in group D only, not in EPL; and two plasma cell clusters, as well one of MO, were only found in group A. Although both FB1 and FB2 were present in group D, FB1 was identified in group C, and FB2 in groups A and B. To distinguish FB1 from FB2, Fb1 was characterized by expressing *HAPLN1*, *IGF2*, *IGFBP3*, *IL6ST*, *OLFML3*, *PHACTR2*, *PITX2*, *S100A10*, *SERPINE2*, and *TGFBI*, but FB2 expresses *COL4A1*, *COL4A2*, *COL6A3*, *COX4I2*, *GATM*, *LAMA2*, *NDUFA4L2*, *NID2*, *PLA2G2A*, and *PPP1R14A*. *HAPLN1* and *S100A10* have been documented among the top 60 expressed genes in villous FB1, FB2, and FB3 [14]. COL4A1, COL4A2, COL6A3, and LAMA2 are the molecules involved in focal adhesion and extracellular matrix pathways associated with premature preterm rupture of membrane (PPROM), which is regulated by lncRNA, as we documented earlier [38]. PVs participate in different phases of chorionic villus vascular development and remodeling associated with placental vasculogenesis [54]. In this study, the marker genes *APOD*, *APOE*, *C1R*, *CFD*, *DCN*, *IGFBP2*, *IGFBP5*, *IGFBP7*, *PTGDS*, and *SPARCL1* were identified in PV1, and *ACTA2*, *HSPB6*, *IFITM2*, *IGFBP2*, *IGFBP4*, *MYL9*, *SELM*, *TAGLN*, *TMSB4*, *XC11* or *f96* in PV2. The lack of PV cell clusters in either sporadic or recurrent miscarriage provided evidence of delayed development of vasculogenesis in EPL.

### Cell development and pathway activity

**Fig. 6:**
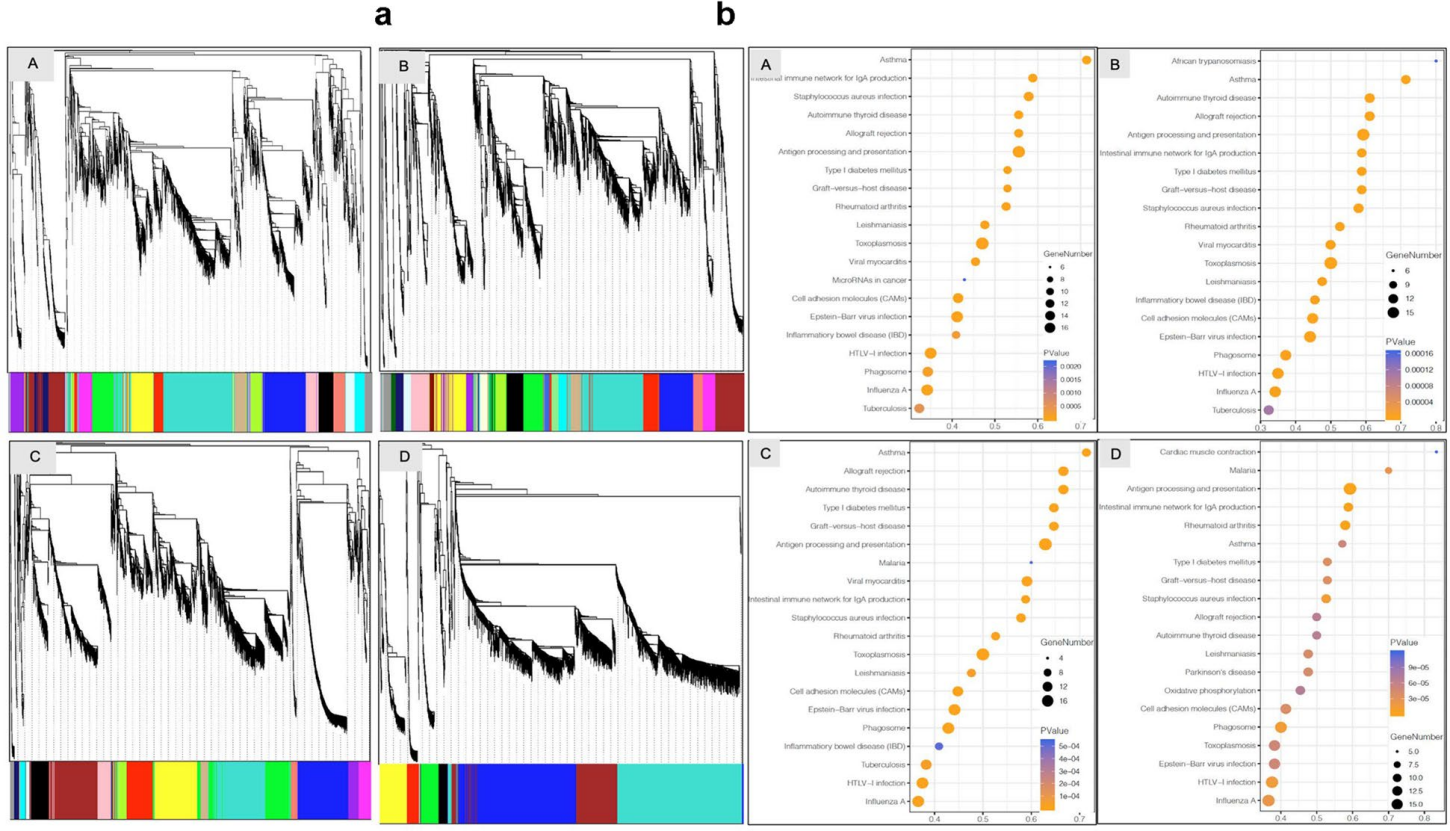
Functional assessment of gene module and pathways. **a** Gene module assignment was assessed with WGCNA in EPL (A_SM, B_SM, and C_RM) and ET (D_EA). The color bar labeled as “Gene Module” beneath the dendrogram represents the module assignment of genes. Each color bar represents the correlation of genes with developmental stage. Red indicates that a gene is positively correlated with a developmental stage and therefore tends to be upregulated at this stage, and green indicates that a gene is negatively correlated with a developmental stage and therefore tends to be downregulated at this stage. **b** Gene set variation analysis (GSVA) showed the top-20 highly enriched pathways, which are associated with human pathological conditions, in the groups of EPL (A_SM, B_SM, C_RM) and ET (D_ET)

Gene co-expression network analysis (WGCNA) [32] was performed for the whole-network connectivity of variably expressed genes among the EPL and ET groups. As shown in Fig. 6a, the WGCNA identified gene modules, each of which may contain a set of genes that are co-expressed at a certain development stage. Pathway enrichment of cell clusters for EPL and ET was assessed with GSVA [55], the gene set variation analysis (Additional file 1: Fig. S3). The top-20 highly enriched pathways characterized in EPL and ET are presented with DotPlot in Fig. 6b. The enriched pathways associated with biological processes, cellular components, and molecular functions are presented in each group of EPL and ET. The highly up-regulated pathways associated with metabolism for VCT cluster are terpenoid backbone biosynthesis in group A, glyoxylate and dicarboxylate metabolism in group B, valine-leucine-isoleucine biosynthesis in group C, and primary immunodeficiency in group D; for STB cluster, citrate-cycle-TCA-cycle in group A, N-glycan biosynthesis in group B, one-carbon-pool by folate in group C, and primary immunodeficiency in group D; and for EVT cluster, mammal circadian rhythm in group A, one-carbon-pool by folate in group B, galactose metabolism in group C, and glycosaminoglycan-biosynthesis-keratan-sulfate in group D. In the fetal macrophage HB cluster, one-carbon-pool by folate is the highly enriched pathway in group A, ganglio-series of glycosphingolipid biosynthesis in group B, glycan degradation in group C, and porphyrin-chlorophyll metabolism in group D. In the maternal macrophage dM cluster, the global series of glycosphingolipid biosynthesis is the highly enriched pathway in group A, primary bile acid biosynthesis in group B, taurine and hypotaurine metabolism in group C, and porphyrin-chlorophyll metabolism in group D. Primary immune deficiency is the enriched pathway in groups A and C, and graft versus host disease is in group B for NK clusters.

**Fig. 7:**
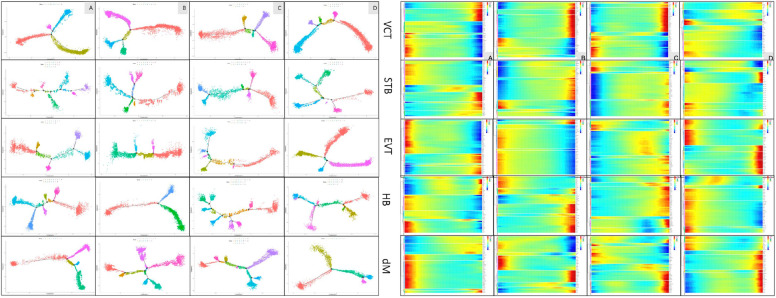
Early development of cell clusters. Pseudotime state (left panel) and pseudotime heatmap (right panel) of trophoblast clusters (VCT1, STB1, and EVT3) and macrophages (HB1 and dM1) in EPL (groups A_SM, B_SM, and C_RM) and in ET (D_ET)

*Characterization of developmental state of placental cell clusters among EPL and ET.* Cellular differentiation and development were plotted with an algorithm of diffusion pseudotime [56]. For the branching trace of trophoblasts, generally, STB showed more branching points, which generated more cell states, but VCT showed fewer branching points in all EPL and ET groups. As presented with Heatmap, the Pseudotime state of differentiation of placental trophoblast clusters are presented in Fig. 7. For macrophages, although there was only one branching point in HB of group B, the higher branching points were shown in group C for both fetal HB and maternal dM, in which there were six branching points that generated 12 cellular states in HB, and five branching points generated nine states in dM.

Based on the gene expression profiles in the cell cycle, as presented by the heatmap illustrated in Additional file 1: Fig. S4, the states of cell cycle stages G1, S, and G2M are presented in a column-bar graphic for each cell cluster [57]. Other than cluster4_FB1 and cluster12_CTB1, the majority of clusters showed that the cell state was at G1 in the group D. In EPL, S stage was in the majority of clusters, except G1 stage was almost 100% in cell clusters dS1 of group B and dM1 of group C.

**Fig. 8:**
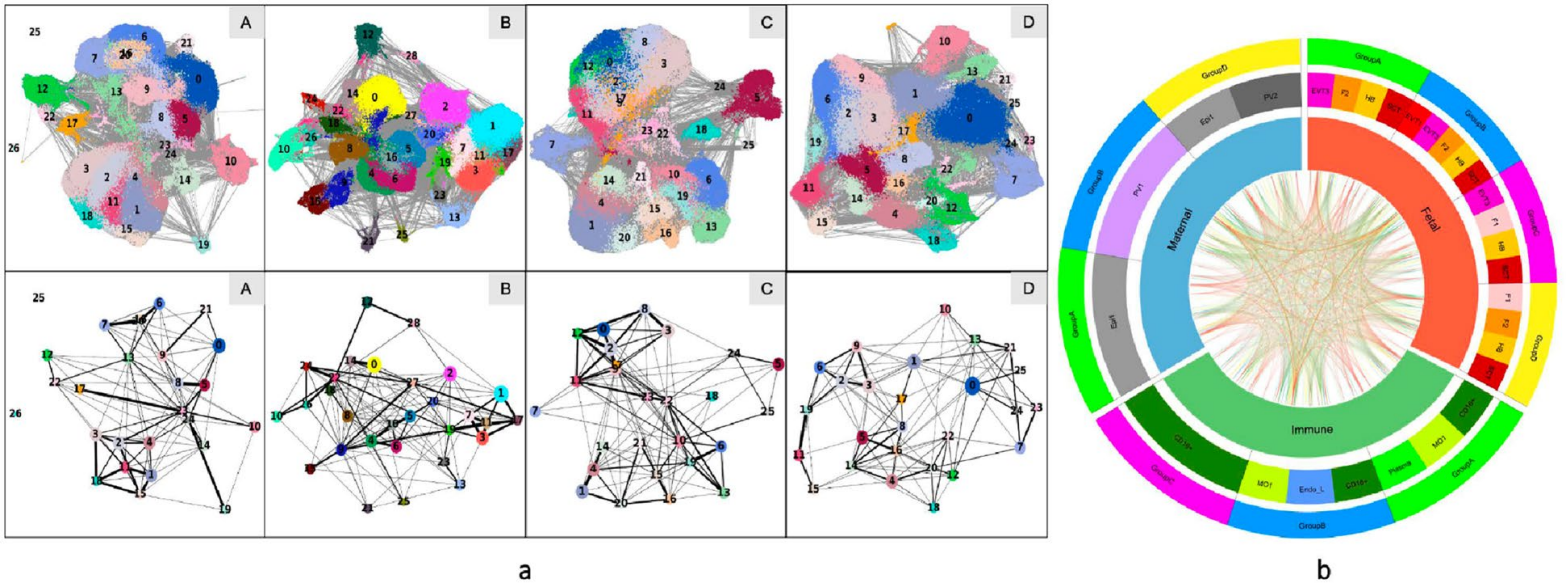
Cell interactions among cell clusters. **a** Cell interactions among cell clusters are represented with tSNE (up) and lines (lower). The number labeled in variant colors matches to the name of clusters illustrated in Fig. 2. **b** Cell interactions among cell clusters of fetal, maternal, and immunological origination are represented by circles. Lines connecting clusters represent the interactions

**Fig. 9:**
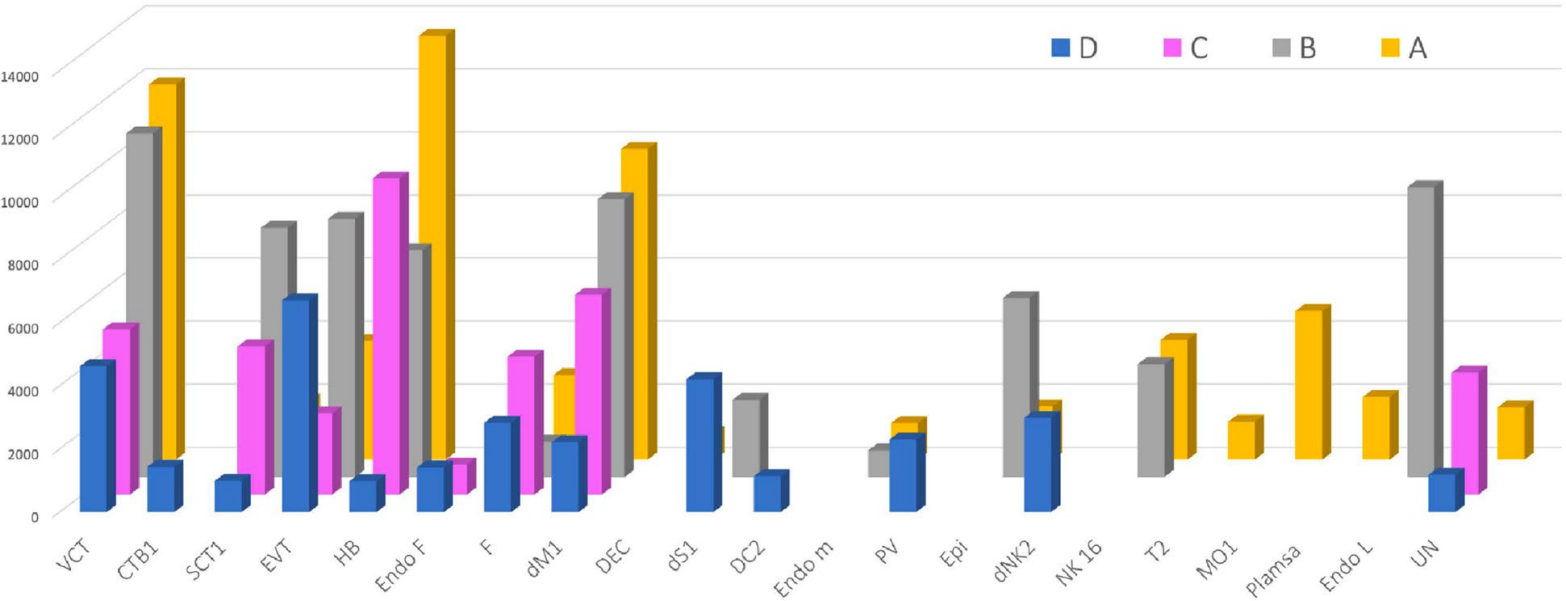
SOD2 gene expression**.** SOD2 gene expression in different cell clusters (X axis) among the four groups of A_SM (orange), B_SM (gray), C_RM (purple), and D_ET (blue)

Scanpy was applied to determine cell–cell interactions [58] (Fig. 8). Cell–cell interaction may be compared in each group of EPL and ET. For example, in group A, VCTs interact with MO1, NK CD16^+^, VCT2, STB, FB2, and EVT3. The interaction between VCT and EVT was mediated through FB2. VCT does not interact with HB in group A; however, it may interact with HB in group C (Fig. 8a). Demonstration of cell–cell interactions may help in exploring the pathophysiological impact of one type of cell on the other type(s) of cells that associate with the development of EPL, as illustrated in Fig. 8b with circles. This was well documented by the differential expression of the gene *SOD2*, which has been found to be expressed highly in trophoblast clusters and in macrophages (both HB1 and dM1). However, *SOD2* expression was relatively low in DC clusters and in blood cell clusters (Fig. 9).

## Discussion

Two of the most critical steps in the establishment of human pregnancy are decidualization and placentation, which are required for maternal interactions with the developing embryo. It is well known that the majority of cases of EPL are the result of chromosomal abnormality [4]. In addition, transcriptomic profiles revealed that the chemokine-cytokine pathway as well as nerve growth factor receptor and SOD2 may present a common pathogenic mechanism associated with EPL, as seen among stillbirths and spontaneous preterm births that we reported recently [51]. The transcriptomic results were generated from bulk RNA sequencing, whereby molecular changes were quantified by estimating the mean value from millions of cells and averaging the signal of individual cells but ignoring cell-to-cell heterogeneity [51]. Therefore, unraveling of the heterogeneity of cell genotype, phenotype, and function within a given subpopulation of placental cells was necessary for a better understanding of the pathogenesis of EPL. In this study, we investigated cell-specific transcriptomics in tissues from EPL via scRNA-seq, with a focus on villous cells, which associate with early placentation, and DCs that associate with decidualization.

EPL has been grouped into three main categories according to causative factors: 1) resulting primarily from villous maldevelopment (such as in aneuploidy and/or lethal fetal anomalies), 2) resulting mainly from abnormal CTB invasion (such as in the antiphospholipid antibody syndrome), and 3) resulting from the abnormalities at the uteroplacental interface (such as in luteal phase deficiency or chronic inflammatory reaction) [59]. Among these three categories, EPL is increasingly considered a placentation disorder, resulting from placental oxidative stress, which may cause a thinner and fragmented trophoblast shell, reduced cytotrophoblast invasion of the endometrium and incomplete plugging of the lumen at the tips of the spiral arteries, and premature onset of the maternal circulation and loss of periphery-center coordination, with blood flow occurring throughout the placenta. Three types of trophoblasts, VCT, STB and EVT, are differentiated from trophectoderm [60–62]. In our earlier study, we discovered that *SOD2,* the mitochondrial superoxide dismutase isoform, was upregulated, being expressed in both fetal and maternal tissues in sporadic miscarriage, in addition to in stillbirth and in spontaneous preterm birth [51]. In this study, we further investigated differential expression of *SOD2* gene in each cell cluster. The increased expression of *SOD2* as a compensatory response to increased generation of superoxide (oxidative stress) in the mitochondria, in the EPL groups, compared to the non-pregnancy loss group D, provided further evidence supporting our results obtained from whole placental transcriptomic studies and indicated that placental oxidative stress is a pathogenic factor involved in the pathogenesis of EPL.

Other than decidual macrophages, in which *SOD2* was expressed in all four groups, four DC clusters—the dS1, DC2, PV, and dNK2—expressed *SOD2* in the group D control group. To our surprise, no *SOD2* was expressed in maternal DC clusters (Fig. 9) or in the blood immune cells, in group C. This result suggested that the pathogenic mechanism associated with group C differs from that of SMs. Indeed, NDUFB3, the NADH dehydrogenase (ubiquinone) 1 beta subcomplex 3 that is a subunit of complex 1 of mitochondrial electron transport chain, has been recently found to be significantly increased in DCs from RM cases. It was documented that overexpression of NDUFB3 may inhibit cell vitality and oxidative stress of DCs and decrease mitochondrial membrane potential expression levels [63]. Decidual CD4^+^T (dCD4^+^T) cells play pivotal roles in inducing and maintaining maternal–fetal tolerance. Functional alteration of cytotoxic T cells (CTLs) in the decidua also contributes to maintaining pregnancies. Dysfunctional dCD4^+^T cells are associated with miscarriage. A decreased number and altered function of Eomes^+^dCD4^+^T cells were observed in miscarriage. A prevalence of CD4 + T cells producing IL-22 and IL-4 (Th17/Th2/IL-22 + , Th17/Th0/IL-22 + , Th17/Th2/IL-22 + , and Th0/IL-22 + cells) was observed in the decidua of successful pregnancies [45, 64]. In this current study, a T cell cluster was identified in the control group of group D, but no T cell cluster was identified in the EPL groups. Whether and how the T cells that have been pathogenically impacted at an earlier stage that resulted in no T cell cluster can be identified from tissues from miscarriage requires further investigation.

In summary, applying scRNA-seq, we have documented variant cell clusters in different subtypes of EPL. Particularly, we determined the marker genes in trophoblasts and in placental macrophages and their development in the early stage of pregnancy and in EPL. Identification of increased expression of *SOD2* in EPL further confirmed that placental oxidative stress is a pathogenic factor throughout the entire period of pregnancy.

## Supplementary Information

**Additional file 1: Figure S1.** Heatmap of marker genes in cell clusters of early pregnancy loss. Heatmap of marker genes in cell clusters of different groups of early pregnancy loss. The numbers of clusters in groups A, B, C, and D were 16, 15, 16, and 18, respectively. **Figure S2.** Dot plot of top-10 marker genes (X axis) in trophoblast cell clusters VCT1 (upper), STB1 (middle), and EVT3 (lower), respectively, among early groups A_SM, B_SM, C_RM, and D_ET. **Figure S3.** Gene set variation analysis (GSVA) analysis. **Figure S4.** Cell cycle

## Data Availability

The datasets, including the Raw and processed data, supporting the conclusions of this article are available in the NCBI Gene Expression Omnibus (GEO; https://www.ncbi. nlm.nih.gov/geo) under accession numbers GSE174399
