# Supplementary material for: Characterization of placental and decidual cell development in early pregnancy loss by single-cell RNA sequencing
Source: Cell Biosci. 2022 Oct 8;12:168. doi: 10.1186/s13578-022-00904-5 (PMC9548121; doi:10.1186/s13578-022-00904-5)
Supplement: Supplementary file 1 — Additional file 1: Figure S1. Heatmap of marker genes in cell clusters of early pregnancy loss. Heatmap of marker genes in cell clusters of different groups of early pregnancy loss. The numbers of clusters in groups A, B, C, and D were 16, 15, 16, and 18, respectively. Figure S2. Dot plot of top-10 marker genes (X axis) in trophoblast cell clusters VCT1 (upper), STB1 (middle), and EVT3 (lower), respectively, among early groups A_SM, B_SM, C_RM, and D_ET. Figure S3. Gene set variation analysis (GSVA) analysis. Figure S4. Cell cycle [file 13578_2022_904_MOESM1_ESM.docx]

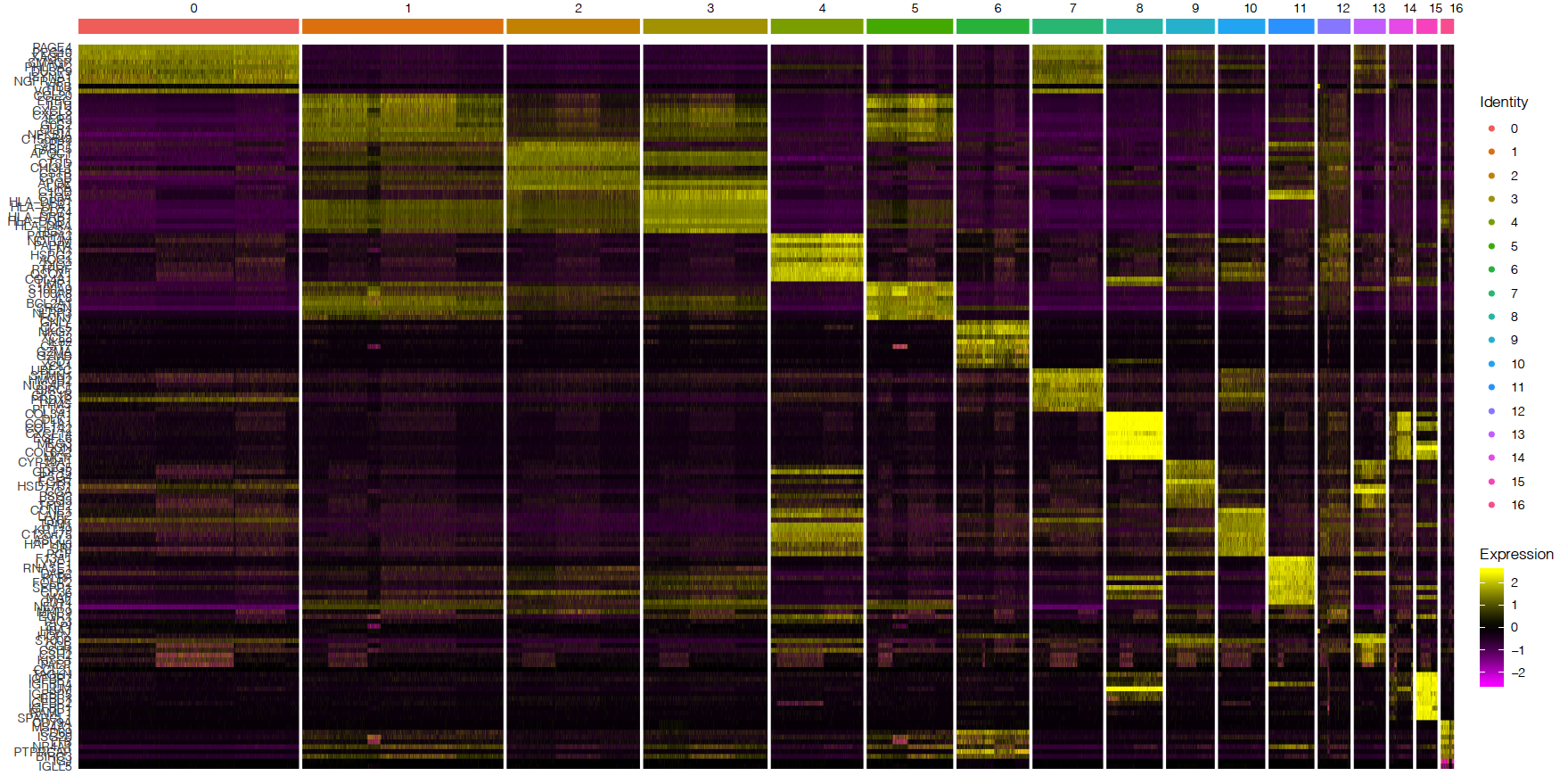

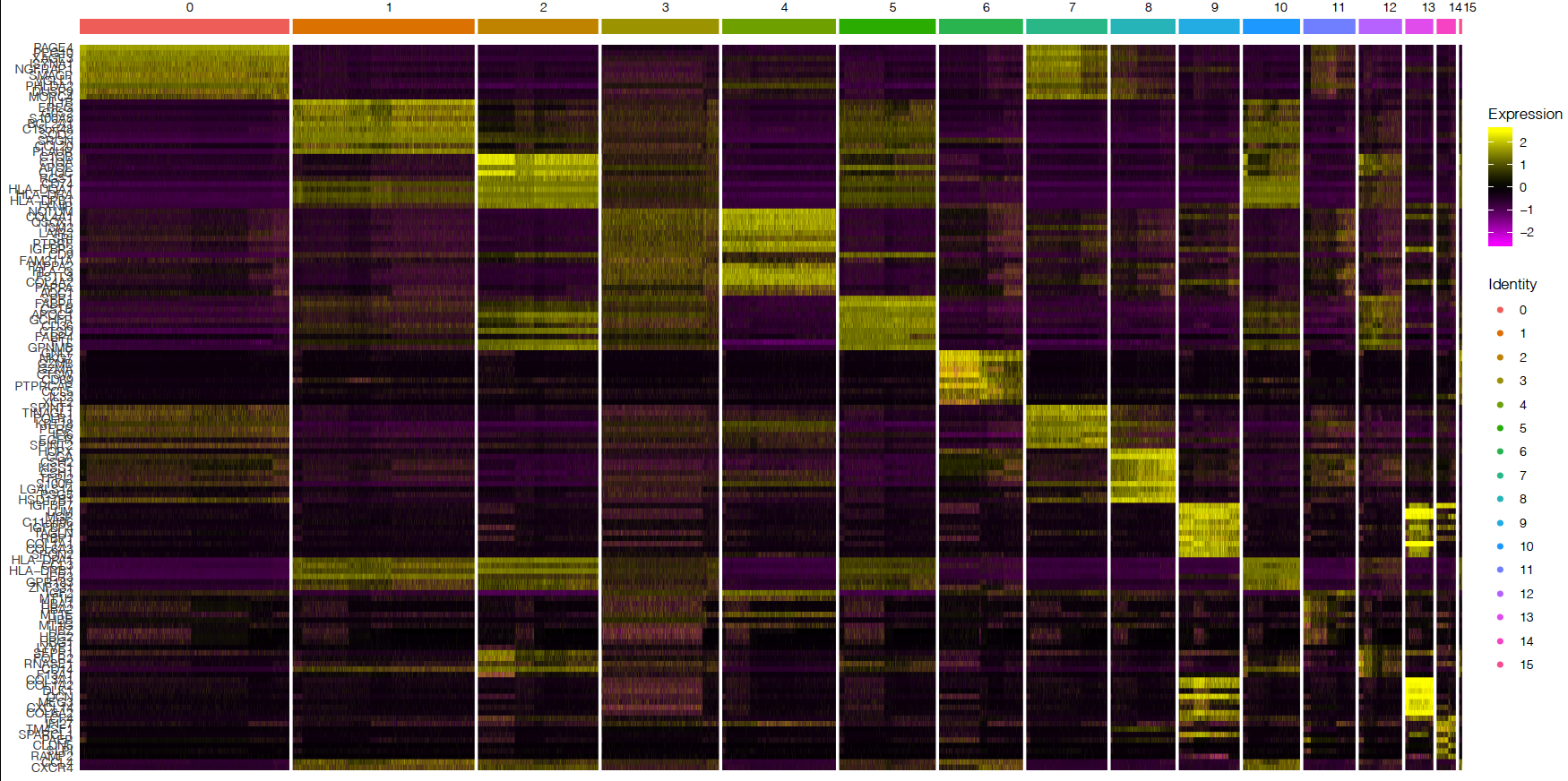

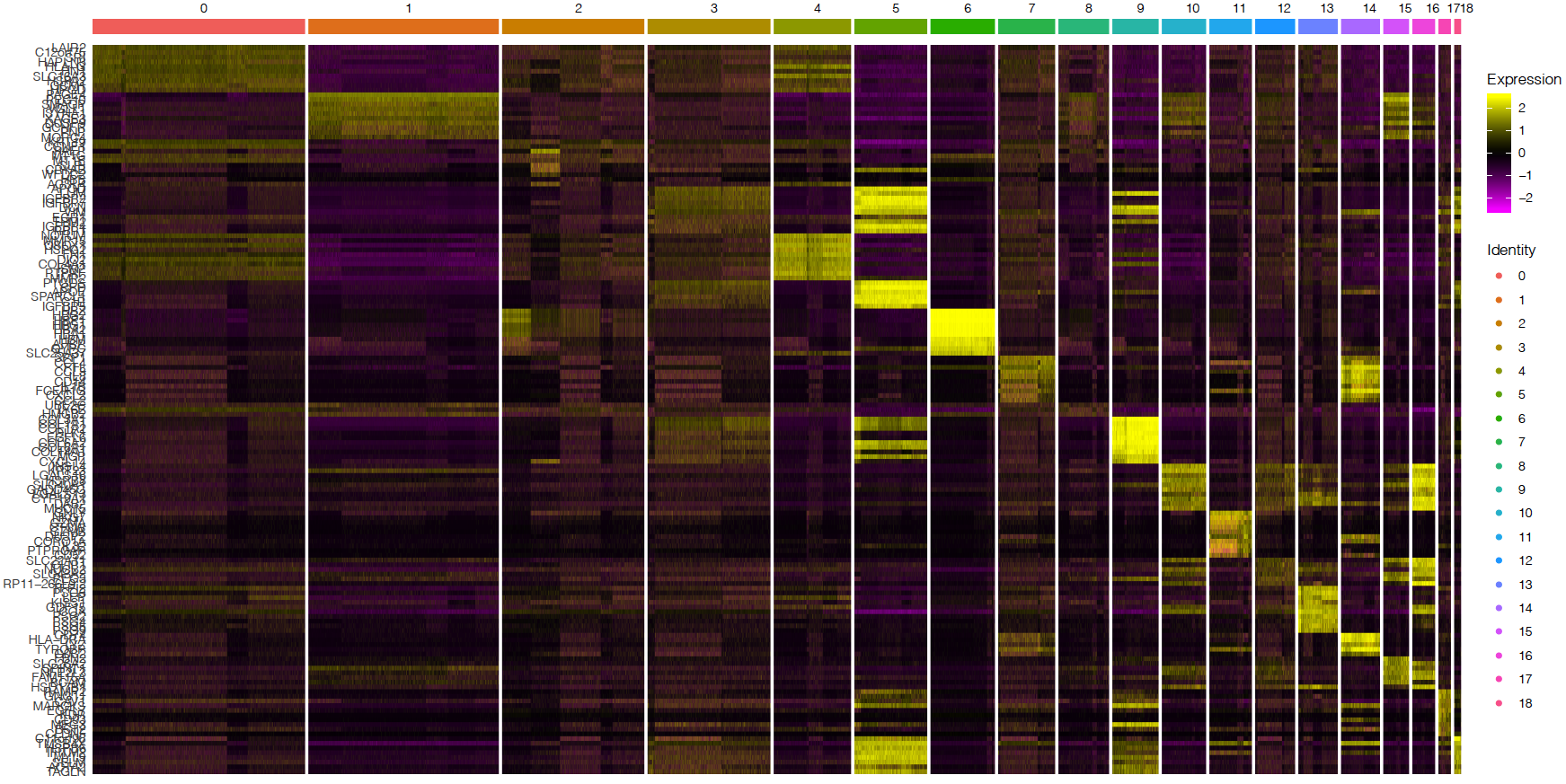


B

D

A


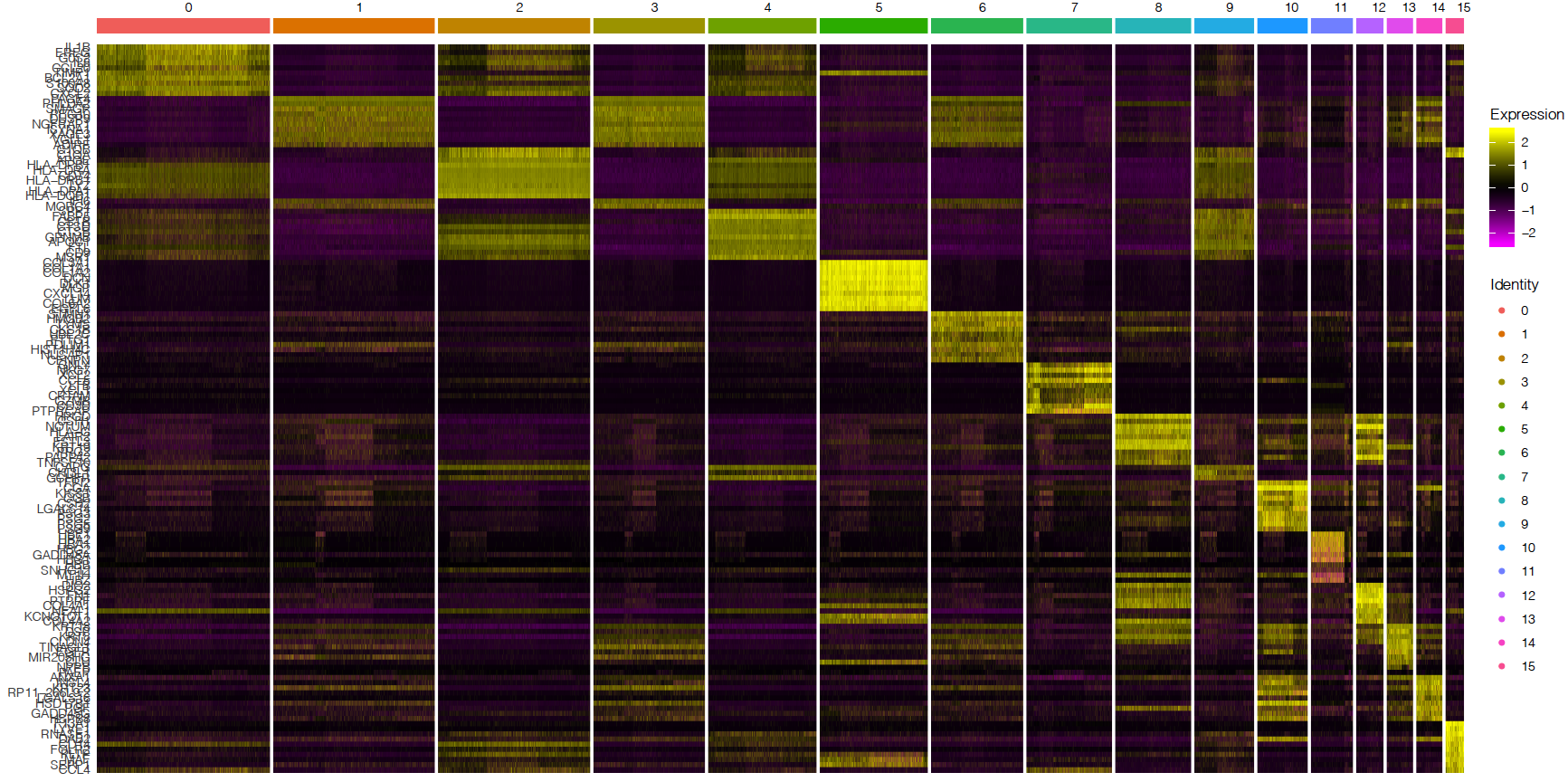


C

**Fig. S1. Heatmap of marker genes in cell clusters of early pregnancy loss.** Heatmap of marker genes in cell clusters of different groups of early pregnancy loss. The numbers of clusters in groups A, B, C, and D were 16, 15, 16, and 18, respectively.


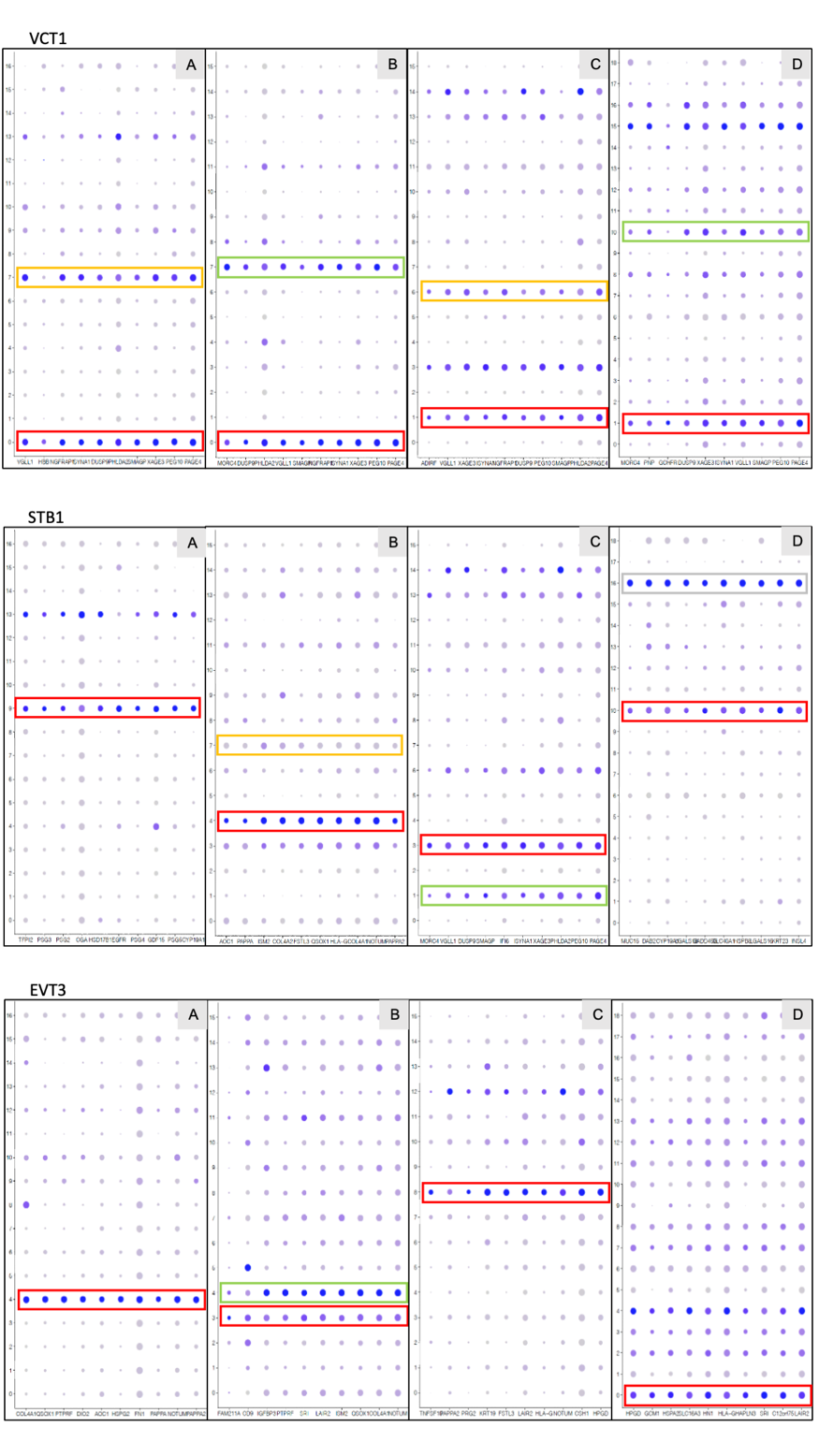


**Fig. S2. Dot plot of top-10 marker genes (X axis) in trophoblast cell clusters VCT1 (upper), STB1 (middle), and EVT3 (lower), respectively, among early groups A_SM, B_SM, C_RM, and D_ET.** Red box presents marker genes expressed specifically in VCT1, STB1, and EVT3, respectively. Orange box shows marker genes expressed in VCT2 (up) or in STB2 (middle). Green box indicates marker genes expressed in VCT1 (middle) or STB1 (lower). A gray box in group D (middle) showed a high expression profile of marker genes for the cell cluster 16, which was labeled as “unidentified” during bioinformatic analysis but appears as a subtype of STB according to the unique marker gene expression profile presented.


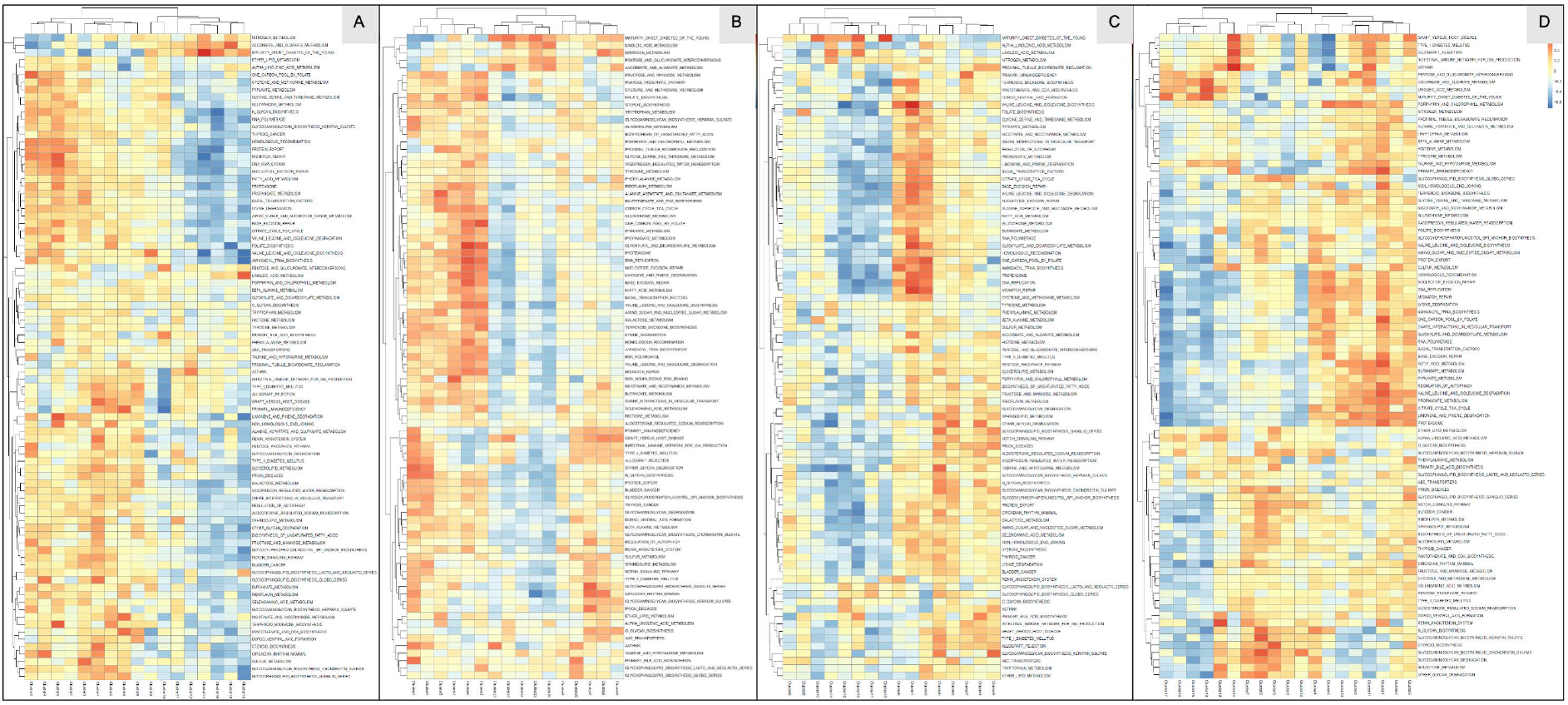


**Fig. S3. Gene set variation analysis (GSVA) analysis.** Pathway activity of variant cell clusters were characterized with GSVA to be different from each other among the groups of EPL (A_SM, B_SM, C_RM) and ET (D_ET).


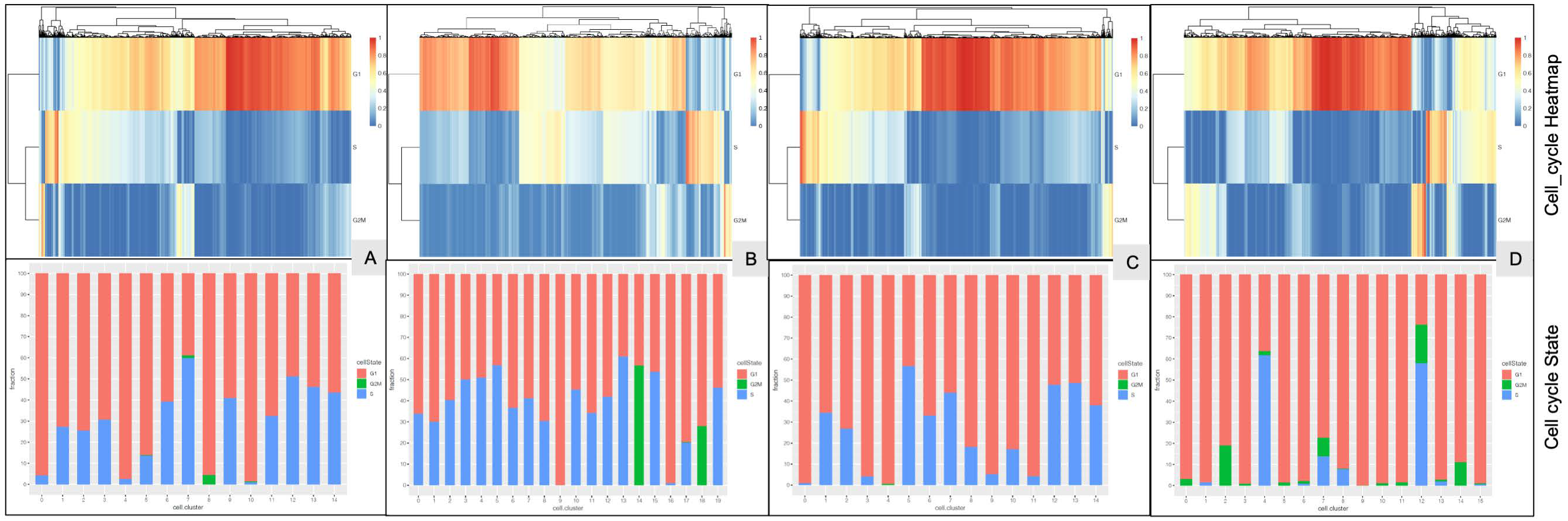


**Fig. S4. Cell cycle.** The cell cycle is represented by heatmap or cell state (red: G1, green: G2M, blue: S), in EPL (groups A_SM, B_SM, and C_RM) and in ET (D_ET).
